# Fabrication of Europium-Doped Barium Titanate/Polystyrene Polymer Nanocomposites Using Ultrasonication-Assisted Method: Structural and Optical Properties

**DOI:** 10.3390/polym14214664

**Published:** 2022-11-01

**Authors:** Umesh Kumar, Diwakar Padalia, Prabhakar Bhandari, Pawan Kumar, Lalit Ranakoti, Tej Singh, László Lendvai

**Affiliations:** 1 Department of Physics, School of Basic & Applied Sciences, K. R. Mangalam University, Gurugram 122103, Haryana, India; 2 Department of Mechanical Engineering, School of Engineering & Technology, K. R. Mangalam University, Gurugram 122103, Haryana, India; 3 Mechanical Engineering Department, Graphic Era (Deemed to be University), Dehradun 248002, Uttarakhand, India; 4 Savaria Institute of Technology, Faculty of Informatics, ELTE Eötvös Loránd University, 9700 Szombathely, Hungary; 5 Department of Materials Science and Engineering, Széchenyi István University, 9026 Győr, Hungary

**Keywords:** barium titanate, polystyrene, XRD, band gap, polymer nanocomposites

## Abstract

In the current work, europium-doped barium titanate particles were used as filler material and polystyrene was used as a matrix to fabricate Ba_1−3*x*_/2Eu_x_TiO_3_/PS polymer nanocomposites with *x* = 0, 0.005, 0.015 and 0.025. A solid-state reaction was used to synthesize filler particles and the solvent evaporation method was used to form polymer nanocomposites. The effects of ultrasonic treatment were also studied in the formation of nanocomposite materials. The quantitative and qualitative studies were conducted using X-ray diffraction (XRD), Fourier transform infrared spectroscopy (FTIR), field emission scanning electron microscopy (FE-SEM), and ultraviolet-visible (UV-Vis) characterization techniques. The XRD data and FTIR data confirm the incorporation of filler particles in the polymer matrix. FE-SEM data confirms that the particles are in the nanophase. The optical band gap was directly affected by the filler particles and it started to reduce as Eu concentration started to increase.

## 1. Introduction

Polymer-based nanocomposite materials have attracted considerable attention from researchers due to their scientific and industrial applications. The introduction of inorganic fillers into the host polymer matrix alters the physical properties of composite material, including electrical, optical, mechanical, etc. These materials have proven their importance in the manufacturing of batteries, capacitors, field-effect transistors, pulsed power systems, quantum dots, etc. [1,2,3,4]. The physical properties of these applications depend upon crystalline structure, temperature and synthesis method [5]. It was also reported that the energy storage performance of polymer composite thin films can be enhanced over a wide temperature range [6]. Polymer structures, like other materials, can be either amorphous or semi-crystalline depending on how they were formed. Polystyrene (PS) has gained scientists’ attention because of its amorphous structure, unique characteristics, outstanding physical and chemical capabilities, and inexpensive cost. Its stiffness, optical transparency, high refractive index, decent electrical insulation, low moisture absorption, and easy colouring and processing make it suitable for a wide range of applications. Polystyrene thin films are brittle and difficult to handle when not supported by a substrate. Tang et al. [7] reported that permittivity and dielectric strength of polystyrene can be improved by nitration and it is possible to achieve high performance polymer dielectrics. Barium titanate (BT) is one of the ceramic materials that has been extensively used as a filler material due to its high dielectric constant, ease of doping, and flexibility to alter characteristics through the optimization of grain size, shape, and dimensions [8]. Ding et al. [9] reported that the dielectric and energy storage properties of barium titanate in a polymer matrix can also be controlled by surface modification. Organic electronic devices require insulating layers with a high dielectric constant and sufficient mechanical flexibility. These requirements can be fulfilled by using barium titanate fillers [10]. Researchers’ interest in barium titanate is growing because of its dependence on crystallographic phases. At the lattice site of the barium titanate structure, dopant ions of various sizes may be accommodated. In order to produce a blend of quality, this quality of adaptation of different dopants at different lattice sites is critical. Barium titanate has gained considerable interest over the past few decades as it might significantly alter optical and other physical characteristics by modifying synthesis routs, introducing dopants at different sites, or changing the particle size. As BaTiO_3_ is a suitable host material for rare earth and transition metal ions, it is a low-cost insulator; that is, it is thermally and chemically stable. Rare earth metal ions, particularly lanthanides, are key luminescence centers. Eu^2+^-doped phosphors have been extensively studied [11]. Rare earths show good stability in the crystal structure of ABO_3_ type perovskites and can enrich the performance of the material. The introduction of rare earths in barium titanate causes grain growth suppression, better temperature response of the dielectric constant, and better reliability in resistance degradation [12,13,14]. The doping of rare earths causes crystal structural distortion, which reflects in terms of change in cell volume, particle size constriction, and inherent strain. These parameters are directly related to the optical, mechanical, dielectric, and electrical properties of filler. Consequently, physical properties can be observed by studying structural properties.

The optical band gap (*E_g_*) of polymer nanocomposites is a critical characteristic that defines the optoelectronic uses of these materials [15,16]. UV-visible (UV-Vis) absorption spectroscopy is commonly used to find the energy band gap of materials and it is predominantly useful for this purpose [17,18]. Due to the low scattering in solid films, it is straightforward to derive the *E_g_* values from their absorption spectra by determining their thickness. Since the greater surface area of colloidal samples is exposed to the light beam, the scattering effect is amplified in such samples. In normal incidence mode, the method (optical absorption) does not distinguish between light that is scattered and absorbed when it is used. This is true for innovative materials that are used in a variety of applications, including aircraft, undersea, automotive, and supercapacitors. There are many new opportunities for advancements in science and technology when polymers are used as the matrix for different types of nano-inclusions.

The purpose of this work is to investigate the structural and optical properties of europium-doped barium titanate polystyrene polymer nanocomposite. The study includes the effects of ultrasonication treatment in the synthesis process. The effect on the optical band gap of polystyrene after loading with different percentages of europium doped barium titanate was also studied.

## 2. Experimental Details

### 2.1. Materials Used

Barium carbonate (BaCO_3_, purity 99.0%), europium (III) oxide (Eu_2_O_3_, purity 99.9%), and titanium (IV) oxide (TiO_2_, anatase, purity 99.0%) were purchased from Sigma-Aldrich (St. Louis, MO, USA), as the first ingredients in the experiment. Fisher Scientific supplied the dichloromethane (CH_2_Cl_2_, purity 99.6%) and the ethanol (ACS grade) was supplied by HiMedia Chemicals (Mumbai, India). Polystyrene ((C_8_H_8_)n, M.W. 13,000) was purchased from Fisher Scientific (Hampton, NH, USA). The powders were dried in a hot air oven at 90 °C for twelve hours before use.

### 2.2. Thermogravimetric Analysis

**Figure 1:**
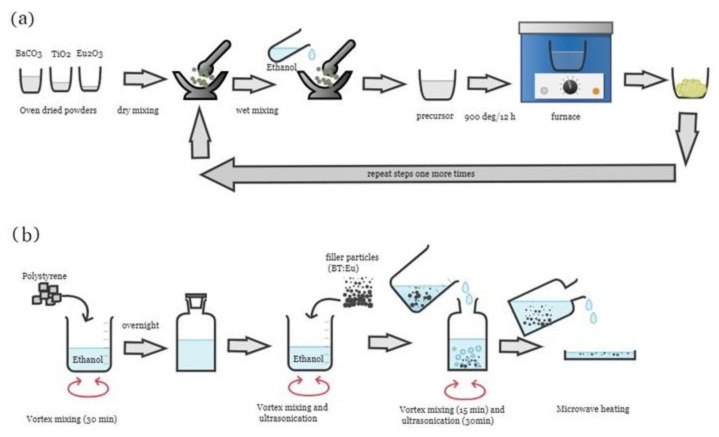
Schematic description of the fabrication steps of (**a**) BaTiO_3_:Eu filler and (**b**) BaTiO_3_:Eu/PS polymer nanocomposites.

The barium titanate powder was prepared using a typical solid-state reaction technique. It was reported that the BaTiO_3_ powders can be synthesized at a lower calcination temperature by using the anatase phase of TiO_2_. In a mortar and pestle, oven-dried powders of BaCO_3_, Eu_2_O_3_, and TiO_2_ were ground together stoichiometrically for 20 min in a dry state. After dry mixing, ethanol was added to the dry mixture and ground again until an almost-dry powder mixture was obtained. To remove any leftover ethanol from the precursors, the mixture was transferred to a borosilicate glass petri dish and dried at 50 °C for six hours. Then, the oven-dried precursors were calcined in a programmable electric furnace at 900 °C for 12 h with a constant heating rate of 5 °C/min. After 12 h of calcination, the samples were cooled at a rate of 5 °C/min. Finally, white lumps of pure and doped barium titanate were formed. The obtained powders were ground again in the dry and wet state using a mortar and pestle. Then, the process of oven-drying and calcination was repeated, as mentioned in the previous step (Figure 1a). After repetition, the final white products obtained were labeled as BaTiO_3_, BaTiO_3_:0.5%Eu, BaTiO_3_:1.5%Eu, and BaTiO_3_:2.5%Eu with a doping level of *x* = 0, 0.005, 0.015, and 0.025.

In order to prepare the polymer nanocomposites, the solvent evaporation method was applied. Polystyrene was used as the matrix and Eu-doped BT was used as the filler phase. The ratio of filler particles relative to the polystyrene matrix was 1:10 (*w*/*w*). To prepare the polymer nanocomposite (Figure 1b), 1000 mg of polystyrene was added to 20 mL of dichloromethane (DCM). The mixture remained like this for 4 h. Then, it was homogenized using a vertex shaker for 30 min and stored in a sealed container overnight. In the next step, 100 mg of pure BT was added to 5 mL ethanol and homogenized using the vertex shaker for 15 min. Then, the solution was ultrasonicated (40 kHz, 140 watt) in an ultrasonic bath for 30 min at room temperature under continuous sonication. This filler mixture was subsequently added to the polystyrene mixture and homogenized using the vertex shaker for 15 min followed by ultrasonication for 30 min. Next, the mixture was transferred to a petri dish and left for 2 h at room temperature. Finally, the mixture was treated with microwaves (2.45 GHz) at 700 watts of power to fabricate the polymer nanocomposite. This sample was labeled as BaTiO_3_/PS. Microwave heat treatment uniformly heated the sample for polymerization (Figure 1b). Following this, the nanocomposite sample was ground into a fine powder. This specimen was labeled as BaTiO_3_/PS. The same technique was repeated to prepare three more samples using the same amounts of PS and Eu-doped BT filler. These samples were labeled as BaTiO_3_:0.5%Eu/PS, BaTiO_3_:1.5%Eu/PS, and BaTiO_3_:2.5%Eu/PS, respectively. It should be noted that the loading level of filler particles in all samples was 10% *w*/*w*.

### 2.3. Characterizations

X-ray diffraction data was recorded on a RigakuUltima IV X-ray diffractometer with Cu Kα radiation. The X-ray diffraction (XRD) patterns were obtained for a 2-theta angle range of 10° to 70° using a speed of 2°/min. QualX 2.24 software was used to analyze the samples qualitatively [16]. Quantitative analysis of XRD data was performed using Expo software. The chemical structure analysis was performed by using a Fourier transform infrared spectrometer (FTIR) (Perkin-Elmer, Spectrum Two, Waltham, MA, USA). The data was recorded in the range of 400–4000 cm^−1^. The morphological study of the polymer nanocomposites was conducted using a JEOL 7610F Plus field emission scanning electron microscope (FE-SEM). The grain size distribution was estimated from the FE-SEM micrographs using the ImageJ image analysis software. To investigate the optical energy band gap using the Shimadzu UV1800 spectrophotometer (Kyoto, Japan), all optical absorption data were collected at room temperature from 200 nm to 800 nm. UV-Visible absorption data and Tauc plot were studied with the help of the Origin software (Version 2021. OriginLab Corporation, Northampton, MA, USA).

## 3. Theoretical Aspects

### 3.1. Lattice Parameters

In ABO_3_-type perovskite structures, BO_6_ octahedra are formed and this octahedral cavity is occupied by smaller cation B. All of the BO_6_ octahedra must be connected through their vertices, and the ratio of the A–O to B–O bonds must be $\sqrt{2}:1$ for an undistorted cubic structure. A variety of models were put forward in order to establish a connection between the ionic radii of A- and B-site cations, anions, and the lattice constants of the cubic and pseudocubic perovskite. 

For the lattice distortion, Ye et al. [19] proposed taking into account not only the cation and anion radii, but also the chemical bonds, partial covalence, and coordination number. The formula for the proposed lattice constant is as follows:

(1) $$a_{\mathrm{Ye}}^{\mathrm{Th}}=0.3166r_{A}+1.422r_{B}-0.1708X_{A}+0.0562X_{B}-0.0066\left(Z_{B}-Z_{A}\right)+2.706$$

where *r_A_* and *r_B_* represent the Shannon ionic radii, *X_A_* and *X_B_* represent the electronegativity, and *Z_A_* and *Z_B_* represent the valency of the A- and B-site ions, respectively.

Moreira et al. [20] proposed a model for the calculation of lattice parameters, as given below:

(2) $$a_{\mathrm{Mor}}^{\mathrm{Th}}=\left[1.8218\left(r_{B}^{\mathrm{VI}}+r_{O}^{\mathrm{II}}\right)+1.1359\;\left\{\frac{\left(r_{A}^{\mathrm{XII}}+r_{O}^{\mathrm{II}}\right)}{\sqrt{2}\left(r_{B}^{\mathrm{VI}}+r_{O}^{\mathrm{II}}\right)}\right\}-0.7785\right]$$

where $r_{A}^{\mathrm{XII}}$, $r_{B}^{\mathrm{VI}}$ and $r_{O}^{\mathrm{II}}$ are the Shannon ionic radii of the A-site cation, B-site cation, and oxygen anion for the corresponding coordination numbers 12, 6, and 2, respectively.

Ubic et al. also proposed a model to link ionic radii and lattice constant using the linear regression technique [21,22], which is stated as follows:

(3) $$a_{\mathrm{Ubic}}^{\mathrm{Th}}=0.06741+0.49052\left(r_{A}^{\mathrm{VI}}+r_{O}^{\mathrm{VI}}\right)+1.29212\left(r_{B}^{\mathrm{VI}}+r_{O}^{\mathrm{VI}}\right)$$

The Equations (1) to (3) can be rewritten in accordance with the stoichiometry (Ba_1−3*x*/2_Eu_x_)TiO_3_/PS polymer composites as follows:

(4) $$\begin{array}{ll} a_{\mathrm{Ye}}^{*\mathrm{Th}} & =0.3166\left[\left(1-\frac{3x}{2}\right)r_{\mathrm{Ba}}^{\mathrm{XII}}+{\mathrm{xr}}_{\mathrm{Eu}}^{\mathrm{XII}}\right]+1.422r_{\mathrm{Ti}}^{\mathrm{VI}}\\ & \;-0.1708\left[\left(1-\frac{3x}{2}\right)X_{\mathrm{Ba}}+xX_{\mathrm{Eu}}\right]+0.0562X_{\mathrm{Ti}}\\ & \;-0.0066\left(Z_{\mathrm{Ti}}-Z_{\mathrm{Ba}}\right)+2.706 \end{array}$$

(5) $$a_{\mathrm{Mor}}^{*\mathrm{Th}}=\left[1.8218\left(r_{\mathrm{Ti}}^{\mathrm{VI}}+r_{O}^{\mathrm{II}}\right)+1.1359\;\left\{\frac{\left(\{\left(1-3x/2\right)\;r_{\mathrm{Ba}}^{\mathrm{XII}}+{\mathrm{xr}}_{\mathrm{Eu}}^{\mathrm{XII}}\}+r_{O}^{\mathrm{II}}\right)}{\sqrt{2}\left(r_{\mathrm{Ti}}^{\mathrm{VI}}+r_{O}^{\mathrm{II}}\right)}\right\}\;-0.7785\right]$$

(6) $$a_{\mathrm{Ubic}}^{*\mathrm{Th}}=0.06741+0.49052\left[\left\{\left(1-3x/2\right)\;r_{\mathrm{Ba}}^{\mathrm{VI}}+{\mathrm{xr}}_{\mathrm{Eu}}^{\mathrm{VI}}\right\}+r_{O}^{\mathrm{VI}}\right]+1.29212\left(r_{\mathrm{Ti}}^{\mathrm{VI}}+r_{O}^{\mathrm{VI}}\right)$$

Table 1 provides a summary of the expected lattice constants and experimentally calculated lattice constants for a variety of compositions (*x* = 0; 0.005; 0.015; and 0.025).

### 3.2. Tolerance Factor

The stability of such ABO3 structures is determined by the lengths of the A–O and B–O bonds. Goldschmidt initially presented the notion of perovskite structural stability in terms of a geometrical ratio known as the Goldschmidt tolerance factor [23].

(7) $$t_{G}=\frac{\left(r_{A}+r_{O}\right)}{\sqrt{2}\left(r_{B}+r_{O}\right)}$$

A stable perovskite structure occurs with a *t_G_* value between 0.81 and 1.11. When pushed beyond this range (0.81 ≤ *t_G_* ≤ 1.11) the cubic or pseudocubic structures become deformed or maybe even destroyed [24]. For a perfect cubic structure, *t_G_*~1. In the event of the value of *t_G_* < 1, slanted corner-sharing octahedra will be reported. On the other hand, linked corner and face sharing phases will begin to occur in ABO_3_-type perovskites whenever *t_G_* > 1. However, with regard to how tightly the ions in the A- and B-sites are packed together, if it needs to be considered, fractional tolerance factors (*t*_1_ and *t*_2_) may be used [25].

(8) $$t_{1}=\frac{\sqrt{2}\left[r_{A}+r_{O}\right]}{a}$$

(9) $$t_{2}=\frac{2\left[r_{B}+r_{O}\right]}{a}$$

Table 1 includes the Goldschmidt tolerance factor (*t_G_*) values and the fractional tolerance factor (*t*_1_ and *t*_2_) for various compositions (*x* = 0, 0.005, 0.015, and 0.025).

## 4. Results and Discussion

### 4.1. X-Ray Diffraction Analysis

**Figure 2:**
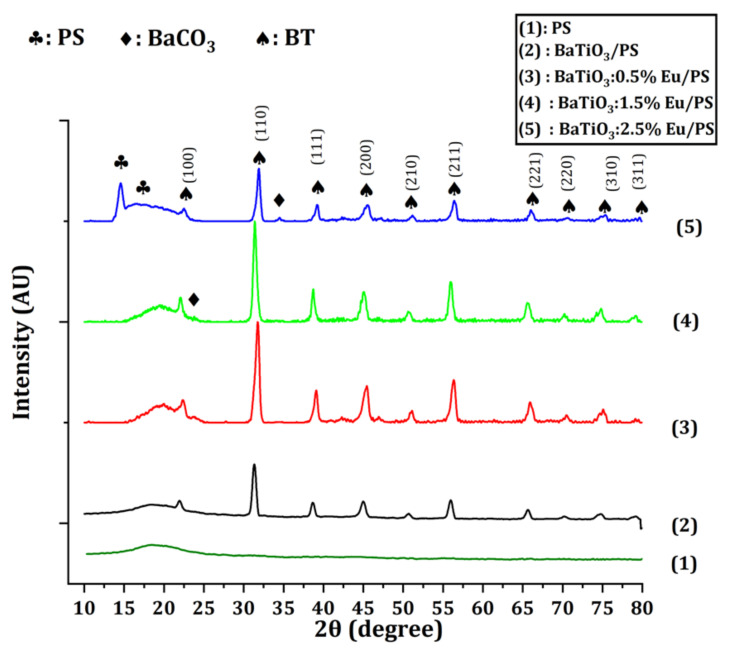
Powder X-ray diffraction (XRD) pattern.

The X-ray diffraction patterns of the europium-doped barium titanate/polystyrene polymer nanocomposites are shown in Figure 2. A distinctive feature of the pattern is the appearance of halos that stretch across the diffraction angle from 14° to 24°. The presence of halos indicates that the amorphous phase predominates in the composite samples. The phase identification of the samples was performed using QualX 2014 (2.24) and the ICDD (PDF2) database was used as the primary database. The QualX analysis reveals that the peaks of both the pure barium titanate-filled composite and europium-doped barium titanate-filled composites were in full agreement with the previously published data. Figure 2 shows the traces of BaCO_3_ (ICDD card no. 00-001-506) for *x* = 0.015 and 0.025. The XRD data also confirms the presence of polystyrene (ICDD card no. 00-013-0836). Traces of BaCO_3_ were also present in filler particles as demonstrated by the XRD of filler particles (Supplementary data Figure S1). These traces formed due to the reaction between barium orthotitanate and carbon dioxide.

$${\mathrm{Ba}}_{2}{\mathrm{TiO}}_{4}+{\mathrm{CO}}_{2}\to {\mathrm{BaTiO}}_{3}+{\mathrm{BaCO}}_{3}$$

The average crystallite size *D* of the filler particles in the polymer matrix was computed using the Scherer formula. The average crystallite size of all predominant peaks was calculated as [17]:

(10) $$D=\frac{K\lambda }{\beta \cos\theta }$$

where *K* is a constant and is called shape factor and its value for cubic crystallites is equal to 0.9 and *β* is called full width and half maxima (FWHM). An average “size–strain plot” (SSP) may be a better way to figure out the size–strain parameters of isotropic line broadening than other methods. This technique has the benefit of putting less emphasis on data from reflections at high angles, which is advantageous since accuracy in these cases is often lower. The SSP equation is as follows [26]:

(11) $${\left(d_{\mathrm{hkl}}\cdot \beta_{\mathrm{hkl}}\cdot \cos\theta \right)}^{2}=\frac{k\lambda }{D}\left(d_{\mathrm{hkl}}^{2}\cdot \beta_{\mathrm{hkl}}\cdot \cos\theta \right)+\frac{\varepsilon^{2}}{4}$$

**Figure 3:**
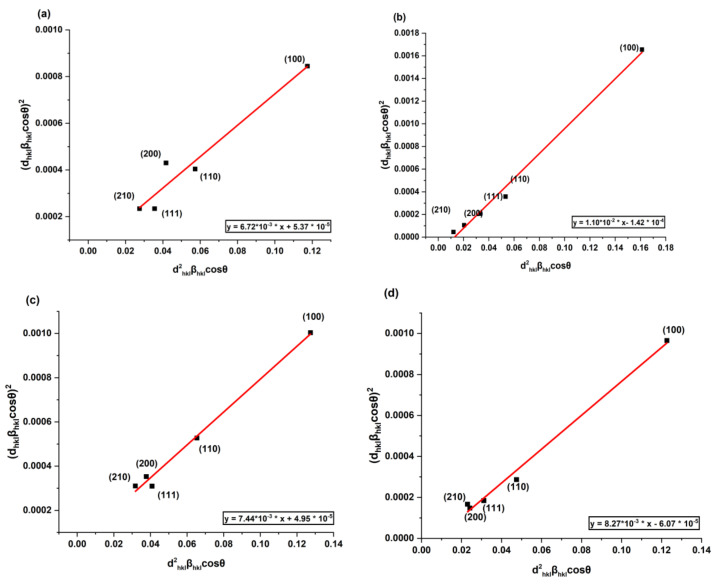
Size-strain plot of (**a**) BaTi_O3_:0.0%Eu/PS.(**b**) BaTi_O3_:0.5%Eu/PS.(**c**) BaTiO_3_:1.5%Eu/PS. (**d**) BaTiO_3_:2.5%Eu/PS.

In comparison, Equation (11) represents the equation of a straight line where ${\left(d_{\mathrm{hkl}}\cdot \beta_{\mathrm{hkl}}\cdot \cos\theta \right)}^{2}$ is referred to as the y–axis and $\left(d_{\mathrm{hkl}}^{2}\cdot \beta_{\mathrm{hkl}}\cdot \cos\theta \right)$ is the x–axis, as shown in Figure 3. The slope of this linear curve gave the average crystallite size, whereas the strain was given by the intercept. Table 1 summarizes all the values of the crystallite sizes that were evaluated using two different approaches: Scherrer formula and size–strain plot. Table 1 also summarizes the variation in strain, lattice parameter, and dislocation density of the filler particles in the polystyrene matrix. 

The source of microstrains may be lattice alteration, size confinement effect, and/or crystal defects. The smaller particle experiences a net force due to the large surface area towards the center, which directly acts on the unit cell. Also, due to the large surface area of nanoparticles, lattice defects are always present, which is another cause of microstrains. Table 1 shows that there is no trend between doping concentration and strain. On the other hand, when comparing the strain and crystallite size of the filler particles in a polystyrene matrix, a trend is found. The strain starts to increase with crystallite size. Thus, the strain in different polymer nanocomposites could be attributed to the particle size effect.

Table 2 shows that cell parameters start to reduce after Eu^+3^ doping in BT. A possible reason for this cell parameter reduction is the size, valency, and electronegativity of the dopant. The ionic radii of Ba^+2^, Ti^+4^, and Eu^+3^ ions are 1.61Å, 0.605 Å, and 1.28 Å, respectively. The radius of the dopant is not a single factor that governs the dopant migration to any site; however, it is also influenced by several other factors, such as electronegativity, charge, crystal structure, etc. The bond length (R) can be calculated as [27]:

(12) $$R=r_{C}+r_{O}-\beta \left(\left|X_{C}-X_{O}\right|\right)$$

where *r_C_* is the covalent radius of the A-/B-site atom, *r_O_* is the covalent radius of oxygen while *X_C_* is the electronegativity of the A-/B-site atom and *X_O_* is the electronegativity of the oxygen atom. The covalent radius (single bond) of the Ba^+2^, Eu^+3^, Ti^+4^, and O^−2^ ions are given by 1.96 Å, 1.68 Å, 1.36 Å, and 0.63 Å, respectively. Therefore, the bond lengths calculated from Equation (12) were found to be 2.360 Å, 2.139 Å, and 1.788 Å for the Ba–O, Eu–O, and Ti–O bonds, respectively. Among these bonds, the Ti–O bond is the smallest and Ti has the highest valency. Thus, due to high Coulombian interaction, high energy is needed to remove the Ti ion from its site. For A-sites, the covalent radii of Ba–O and Eu–O are comparable. Taking the ionic radii of the Ba^+2^ and Eu^+3^ ions into consideration, it is quite reasonable that the Eu^+3^ ions will replace the Ba^+2^ ions. Hence, the reduction of the lattice parameters in Eu^+3^-doped BT/PS polymer nanocomposites (*x* = 0.005 and *x* = 0.015) is related to the reduction of the A–O bond length and the replacement of small ionic radii Eu^+3^ ions with Ba^+2^ ions.

These results were also supported by the tolerance factor data (Table 2). The values of the fractional tolerance factors (Equations (8) and (9) indicate how closely the ions are packed. If the value of the fractional tolerance factors is greater than one, then the ions will be closely packed in lattice, while values lower than one indicate the swaying nature of ions in a site. As the Eu^+3^ concentration increases, the value of *t_2_* approaches unity, except for the concentration *x* = 0.025 (Table 2). This trait shows that the Ti^+4^ ions become more closely packed after doping. Meanwhile, a decrease was observed in *t_G_* and *t_1_* after doping, which indicates that A-site ions become less closely packed after doping. The overall effect reflects a controlled reduction in cell parameters. Comparing the experimental values of cell parameters with theoretical values (Equations (4) and (6)), the experimental and theoretical results are in good agreement with one another. This indicates that theoretical prediction models (Equations (4) and (6)) for cell parameter prediction can be used for filler particles as well as polymer composite material. Dislocation density gives information about the defects in the material and is given by [17]:

(13) $$\delta =1/D^{2}$$

where *D* is the crystallite size (SSP). 

The values of dislocation densities for different polymer composites were summarized in Table 1. The increase in dislocation density is correlated with the increase in disorder and vice versa. The number of the unit cells (*n*) in the particle can be calculated as [28]:

(14) $$n=\frac{\pi D^{3}}{6V}$$

where *V* is the volume of the unit cell and crystallite size (SSP). Table 1 shows the values of the number of unit cells. This datum shows that Eu-doping reduces the nucleation process and reduces the number of unit cells.

### 4.2. Fourier-Transform Infrared (FTIR) Analysis

**Figure 4:**
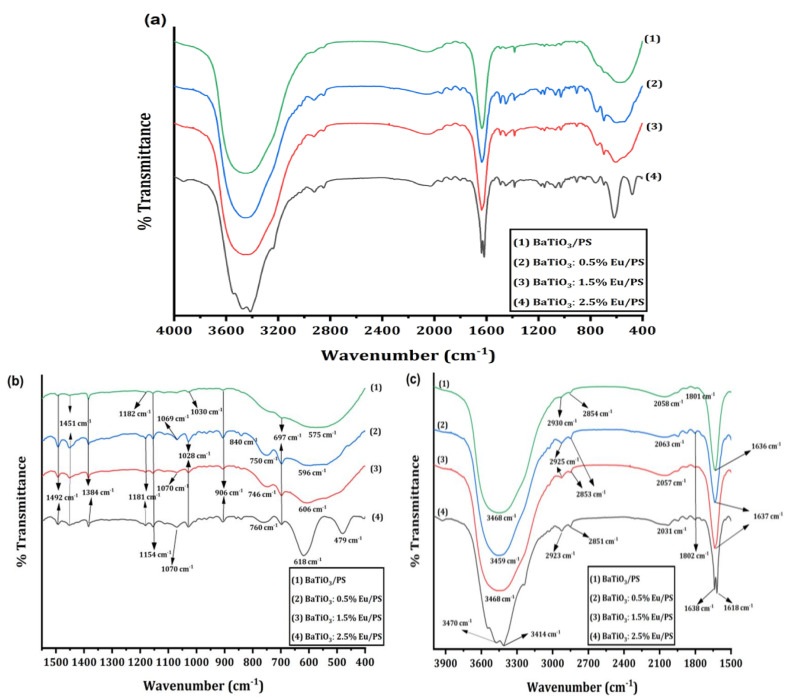
(**a**) Complete FTIR spectra of BaTiO_3_:Eu/PS polymer nanocomposites (**b**) Fingerprint region of BaTiO_3_:Eu/PS polymer nanocomposites (**c**) The functional group region of BaTiO_3_:Eu/PS polymer nanocomposites.

Figure 4a–c illustrates the Fourier-transform infrared (FTIR) spectra of Eu-doped barium titanate polystyrene polymer nanocomposites. The intense bands were found in the range of 400 to 800 cm^−1^, 1200 to 1800 cm^−1^, and 2900 to 3600 cm^−1^. The IR spectra of polymer nanocomposites clearly show the presence of polymeric groups and filler particles. The absorption band region from 650 to 400 cm^−1^ corresponds to the vibrations ofTiO_6_ octahedrons [29]. A strong absorption peak appears at 618 cm^−1^ for BaTiO_3_/PS, which corresponds to Ti–O_I_ stretching normal vibrations of the connected TiO_6_ octahedra. The peak at 479 cm^−1^ represents the bending normal vibrations of the Ti–O_II_ bond. Figure 4b shows that the absorption band in the range of 550–675cm^−1^ gets broader after Eu-doping. This broadening is attributed to the formation of new bonds as a consequence of doping. It should also be noted that the absorption peaks of Eu-doped samples near 600 cm^−1^ have been shifted toward the higher wave number side as the doping level increases. This indicates an increase in the Ti–O_I_ bond energy. From the tolerance factor data, it is clear that the fractional tolerance factor *t_1_* starts to reduce after doping (Table 1). This indicates that A-site ions dangle in their sites. It is also noteworthy that one Ba^+2^ vacancy will be created for two Eu^+3^ ions that are replaced for charge neutrality. This reduces the coordination number of oxygen ions in the Ti–O octahedra. Consequently, the Ti–O bond is contracted, which increases the force constant. The wave number is given by:

(15) $$\bar{\nu }=\left(1/2\pi c\right){\left[k/\mu \right]}^{1/2}$$

(16) $$\mu =\left[\frac{\left\{\left(1-x\right)m_{\mathrm{Ba}}+xm_{\mathrm{Eu}}\right\}m_{O}}{\left\{\left(1-x\right)m_{\mathrm{Ba}}+xm_{\mathrm{Eu}}\right\}+m_{O}}\right]$$

where *k* is force constant and *µ* is reduced mass. Clearly, the wave number depends on reduced mass and force constant. The reduced mass starts to decrease with an increase in Eu concentration. The replacement of Ba^+2^ ions with Eu^+3^ ions enhances the Coulombian force and reduces the overall bond length, which is reflected in cell parameter reduction, as also confirmed by the XRD data. Consequently, the force constant starts to increase with Eu^+3^ doping. The collective effect of an increase in force constant and reduction in reduced mass causes shifting of the wavenumber, near 618 cm^−1^, towards the higher side after doping (Figure 4b). The absorption peaks at 1492 cm^−1^ and 1451 cm^−1^ represent the stretching vibration bands of aromatic ring C=C contribution from polystyrene [30]. The absorption peaks near 2925 cm^−1^ and 2851 cm^−1^ confirm the asymmetric and symmetric stretching vibrations of the methylene group, respectively [31]. The peak at 697 cm^−1^ was attributed to an aromatic ring bend, while the peaks near 746 cm^−1^ were attributed to aromatic out-of-the-plane -CH bending [10]. The peak at 906 cm^−1^ was attributed to the out-of-plane bending mode of the hydrogen atoms in the phenyl ring of polystyrene. The peak at 1638 cm^−1^ represents the bending vibration of molecularly adsorbed water, while a broad band centered near 3468 cm^−1^ is attributed to hydroxyl stretching vibrations. The presence of these bands in all samples confirms the formation of polymer composites.

### 4.3. Morphological Study

**Figure 5:**
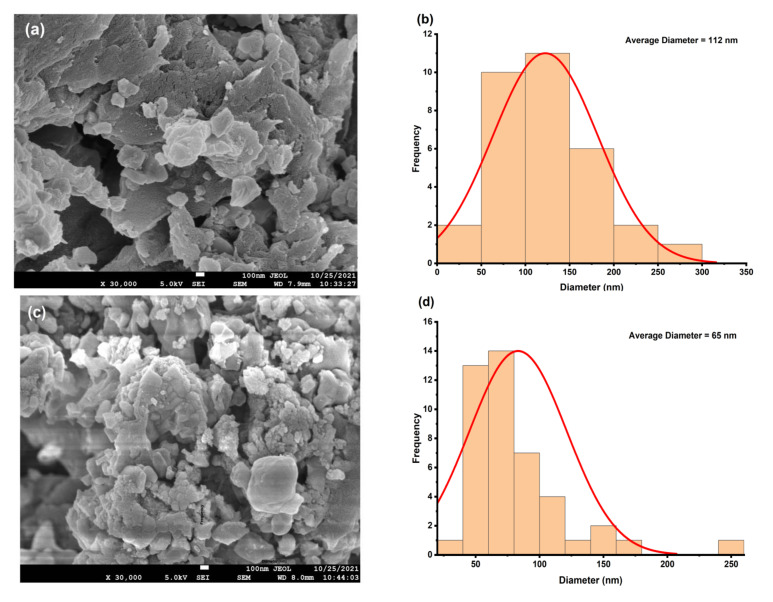

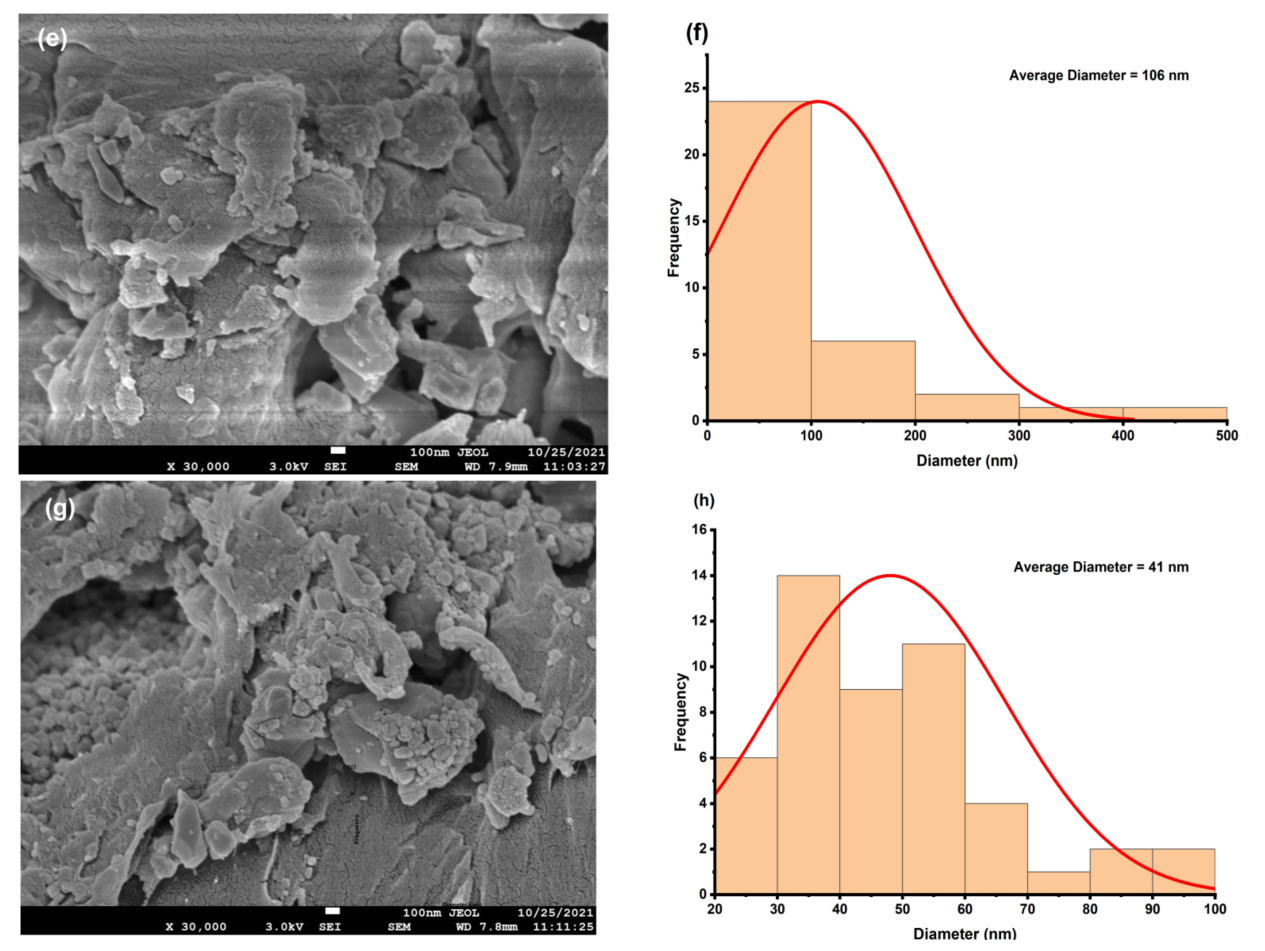
FE-SEM micrographs and particle size distribution. (**a**,**b**) BaTiO_3_/PS polymer nanocomposite. (**c**,**d**) BaTiO_3_:0.5%Eu/PS polymer nanocomposite. (**e**,**f**) BaTiO_3_:1.5%Eu/PS polymer nanocomposite. (**g**,**h**) BaTiO_3_:2.5%Eu/PS polymer nanocomposite.

Figure 5a–d shows the FE-SEM micrographs of pure and Eu-doped BaTiO_3_/PS composite samples. FE-SEM micrographs clearly illustrate that small particles were embedded in polystyrene flakes. In pure BaTiO_3_/PS, the particle size varies from 44 nm to 299 nm with an average size of 112 nm, as shown in the inset of Figure 5a. The average particle size was found in the range 41–106 nm for doped samples (Table 1). A difference between particle size and crystallite size was found in Ba_1−3*x*/2_Eu_x_TiO_3_/PS polymer nanocomposites (Table 1). This result indicates that the particles consisted of several aggregated crystallites. This crystallite agglomeration may be attributed to clamping force. The quantitative estimation of the number of aggregated crystallites can be done by using Equation (14). It should also be noted that the particle size was not too large and maintained around nano-phase for all Ba_1−3*x*/2_Eu_x_TiO_3_/PS samples. Comparing the particle size of filler particles before introducing them into the polymer, it can be found that the particle size of the filler particles ranges from 161 nm to 229 nm (Supplementary data Figure S2). Here we can clearly observe the effect of ultrasonication in the formation of polymer composites. The sonication energy (J/mL) supplied to the composite during the sonication process is given as

*E = P × t/V* (17)

where *P* is power of the sonicator in Watts, *t* is sonication time in seconds and *V* is the volume of the solution that is under the sonication process. Starting synthesis conditions to prepare polymer nanocomposites were 140 W power, 1800 s sonication time, and 25 mL solution volume. Thus, the sonication energy supplied to each sample was 10.08 kJ/mL. A possible reason for this de-agglomeration of filler particles may be acoustic cavitation due to then ultrasonic treatment given to the samples. In the acoustic cavitation process, bubbles form, advance and finally collapse in the medium due to compression and rarefaction. A very high pressure and temperature are generated during the collapse of the bubble. The collapse of the bubble also produces shock waves that may fracture the filler particles. In addition, the cooling rate is also very high, which hinders the agglomeration and crystallization process. Consequently, the particle size obtained after ultrasonication is sufficiently small.

### 4.4. UV-Visible Absorption Analysis

**Figure 6:**
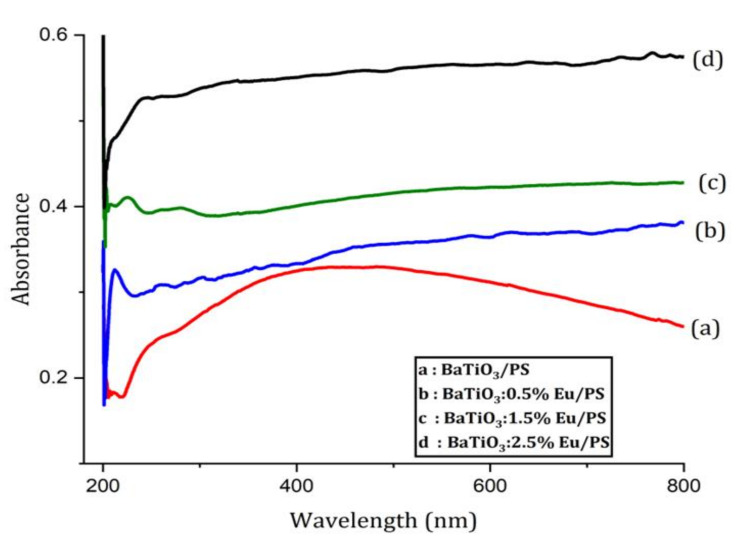
The absorbance spectra for the BaTiO_3_:Eu/PS polymer nanocomposite.

The UV-Vis absorption spectrum is a very important tool for figuring out the optical energy bandgap of both crystalline and amorphous materials. Figure 6 shows the absorption spectra of BaTiO_3_:Eu/PS polymer nanocomposite. The shoulders appeared in the samples in the range 240–280 nm, which may be due to an interaction between the neighboring phenyl rings present in the polystyrene matrix. This rotation of phenyl rings around the C–C bond was due to the flexibility of polystyrene chains. It should also be noted that the absorbance starts to increase as Eu concentration starts to grow. This behavior can be attributed to donor levels created due to Eu doping. As the concentration of Eu starts to increase, more and more donor levels will be introduced and the movement of donor level electrons in the conduction band will increase the absorption. In addition, the absorption spectrum of Eu-doped samples gives a plateau region in the visible region. This result indicates that the BaTiO_3_:Eu/PS polymer nanocomposites may be potential materials for UV filter and UV- shielding applications. 

The character of the electron transition may be somewhat ascertained by the absorption coefficient. In brief, direct and indirect transitions have the potential to take place near the absorption edge of materials. The Tauc relation establishes a connection between the optical band gap (*E_g_*) and the absorption coefficient (*α*) and is given by the following relation:

*αhυ* = *β* (*hυ* − *E_g_*)*^n^* (18)

where *β* and *n* are constant. The absorption coefficient (*α*) is given by 2.303A/t, where A and t stand for absorbance and thickness of the sample, respectively. The exponent ‘*n*’ varies depending on whether the electronic transition is direct or indirect. *n* = 2 or 1/2 is used for indirect and direct allowed transitions [32]. In this current paper, only direct transitions (*n* = 1/2) are considered, so Equation (18) can be written as:

(*αhυ*)^2^ = *β* (*hυ* − *E_g_*) (19)

**Figure 7:**
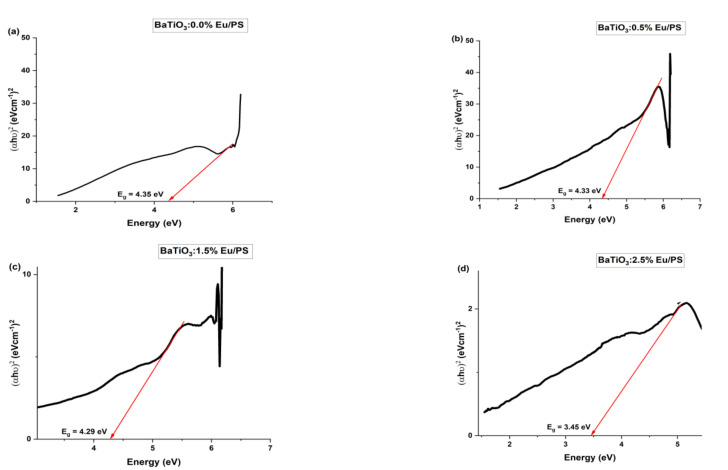
Tauc plot of (**a**) BaTiO_3_/PS. (**b**) BaTiO_3_:0.5%Eu/PS. (**c**) BaTiO_3_:1.5%Eu/PS. (**d**) BaTiO_3_:2.5%Eu/PS.

As shown in Figure 7, the graphs depict the relationship between (*αhυ*)^2^ and photon energy (*hυ*) in order to determine the values of *E_g_* for the analyzed samples. The direct band gap for the Eu-doped barium titanate-polystyrene polymer nanocomposites was computed by extrapolating the linear parts of (*αhυ*)^2^ to zero absorption along the *hυ* axis. According to the findings, the direct optical band gap values of polystyrene were reduced as the percentage of europium-doped barium titanate filler nanoparticles increased (Table 1). A continuous decrease in the energy band was found with increasing Eu-doping concentrations. 

This decrease in the optical band gap can be explained by considering the optical band gap of filler particles and the polystyrene matrix. The optical band gap of polystyrene and barium titanate is 4.4 eV and 3.2 eV, respectively [33,34]. The addition of filler particles in a polystyrene matrix increases the degree of disorder in the matrix and increases the flexibility of polymer chains. The interaction of polymer and filler particles modifies the energy levels of polystyrene and introduces new energy levels of filler. Furthermore, the replacement of Ba^+2^ ions with Eu^+3^ ions creates Ba vacancies as per the Kroger–Vink notation for this substitution:

$${\mathrm{Eu}}_{2}O_{3}\overset{{\mathrm{BaTiO}}_{3}}{\to }2{\mathrm{Eu}}_{\mathrm{Ba}}^{\cdot }+V_{\mathrm{Ba}}^{\prime \prime }+3O_{O}^{\times }$$

This replacement reduces the lattice constant and distorts TiO_6_ octahedra, which was also confirmed by XRD data. The distortion in TiO_6_ octahedra introduces sub-electronic defect levels within the optical band gap and causes a reduction in bandgap energy.

## 5. Conclusions

In summary, various concentrations of europium-doped barium titanate ${\mathrm{Ba}}_{1-3x/2}{\mathrm{Eu}}_{x}{\mathrm{TiO}}_{3}$ (*x* = 0.000, 0.005, 0.015, 0.025) fillers were synthesized via solid state reaction method. These filler particles were successfully incorporated into a polystyrene host matrix by using the solvent evaporation method. The XRD analysis confirmed the presence of crystalline pure and Eu-doped BaTiO_3_ phases, together with the presence of an amorphous polystyrene phase. This datum clearly shows the relationship between crystallite size and induced strain. A high value of strain causes a reduction in crystallite size. The ultrasonic treatment given to the filler particles is beneficial to reduce the particle size of the filler as confirmed by FE-SEM results. 

The optical energy band gap deduced from the Tauc plot shows a decreasing trait as the doping level increases. This decrease in the optical band gap of polymer nanocomposite is devoted to the introduction of filler in a polystyrene matrix. The introduction of filler particles causes localized energy levels in the polymer matrix. The increase in Eu concentration introduces sub-electronic defect levels within the optical band gap of barium titanate, which is the reason for the further decrease in the optical band gap of polymer nanocomposites. Although the band gap is also influenced by particle size and strain, our samples do not show any direct influence of these factors. The absorption spectra of polymer nanocomposites do not contain any peaks in the visible region. This finding suggests that the material might be used as a UV filter or UV-shielding block.

## Figures and Tables

**Table 1:** Crystallite size calculated by Scherrer formula and size-strain approach (SSP) of Ba_1−3*x*/2_Eu_x_TiO_3_/PS (10% *w*/*w*).

Composition	D (Scherrer) nm	D (SSP) nm	d (FESEM) nm	n	δ × 10^−3^ (SSP, nm^−2^)	ε × 10^−2^	Eg (eV)
*x* = 0.000	20.16	20.63	112	71.49515	2.35	1.47	4.35
*x* = 0.005	27.61	12.61	65	16.45934	6.28	2.38	4.33
*x* = 0.015	17.89	18.63	106	53.21427	2.88	1.41	4.29
*x* = 0.025	19.58	16.76	41	38.40016	3.56	1.56	3.45

D—crystallite size, d—particle size, n—number of unit cells per particle, δ—dislocation density, ε—strain, Eg—energy band gap.

**Table 2:** Comparison of lattice parameters calculated from various theoretical approaches with experimental (*a^expt^*) and Goldschmidt tolerance factor (*t_G_*) with fractional tolerance factor (*t_1_* and *t_2_*).

Composition	$a_{\mathrm{Ye}}^{*\mathrm{Th}}$ (Equation (4))	$a_{\mathrm{Mor}}^{*\mathrm{Th}}$ (Equation (5))	$a_{\mathrm{Ubic}}^{*\mathrm{Th}}$ (Equation (6))	$a^{\mathrm{expt}}$ in nm	$t_{G}$ (Equation (7))	*t*_1_ (Equation (8))	*t*_2_ (Equation (9))
*x* = 0.000	3.99737	3.99922	4.00704	4.00559	1.07061	1.0449	0.97614
*x* = 0.005	3.99569	3.99689	4.00440	3.99488	1.06727	1.0446	0.97875
*x* = 0.015	3.99233	3.99222	3.99911	3.99144	1.06445	1.04257	0.9796
*x* = 0.025	3.98896	3.98756	3.99382	4.00334	1.06034	1.03546	0.97668

## Data Availability

The data are available from the first author upon reasonable request.

## Supplementary Materials

The following supporting information can be downloaded at: https://www.mdpi.com/article/10.3390/polym14214664/s1, Figure S1: Powder X-ray diffraction pattern of pure and Eu-doped barium titanate filler particles; Figure S2: FE-SEM micrographs of and particle size distribution (a,b) BaTiO3 (c,d) BaTiO3:0.5%Eu (e,f) BaTiO3:1.5%Eu (g,h) BaTiO3:2.5%Eu.
