# Supplementary material for: Fabrication of Europium-Doped Barium Titanate/Polystyrene Polymer Nanocomposites Using Ultrasonication-Assisted Method: Structural and Optical Properties
Source: Polymers (Basel). 2022 Nov 1;14(21):4664. doi: 10.3390/polym14214664 (PMC9655646; doi:10.3390/polym14214664)
Supplement: Supplementary file 1 [file polymers-14-04664-s001.zip › polymers-1965313-supplementary.pdf]

polymers

MDPI

Supplementary data

[Fabrication of Europium-Doped Barium Titanate/Polystyrene Polymer Nanocomposites Using Ultrasonication-Assisted Method: Structural and Optical Properties]

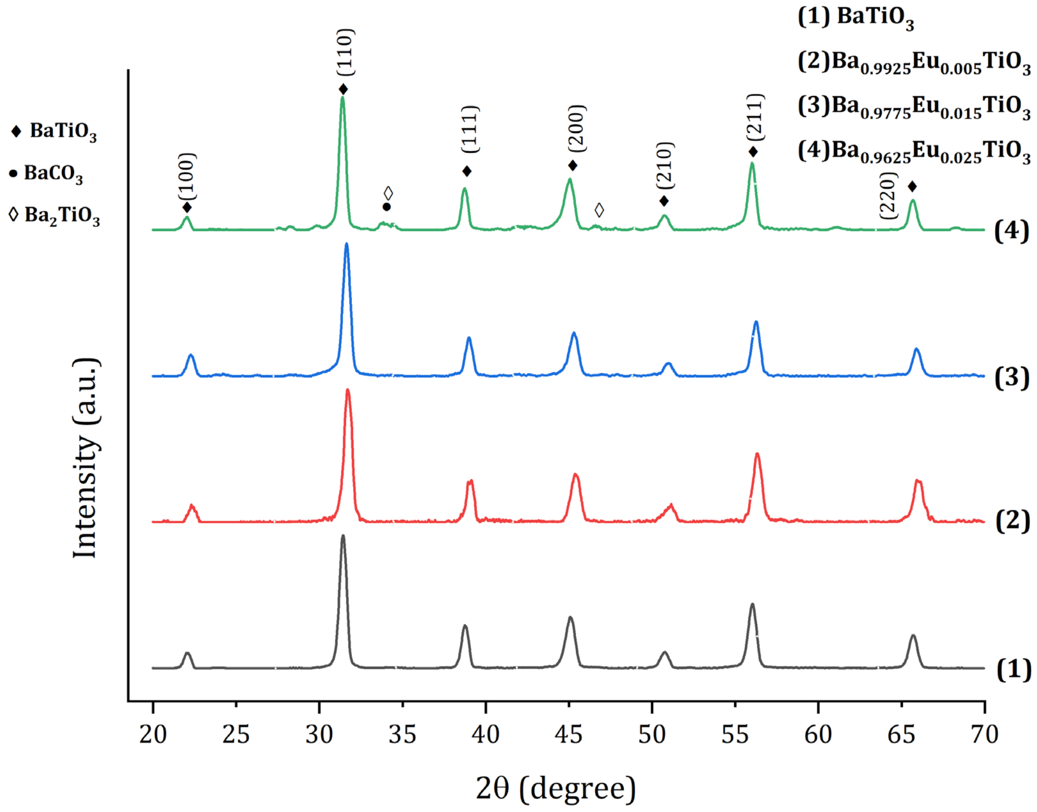

Figure S1. Powder X-ray diffraction pattern of pure and Eu-doped barium titanate filler particles.

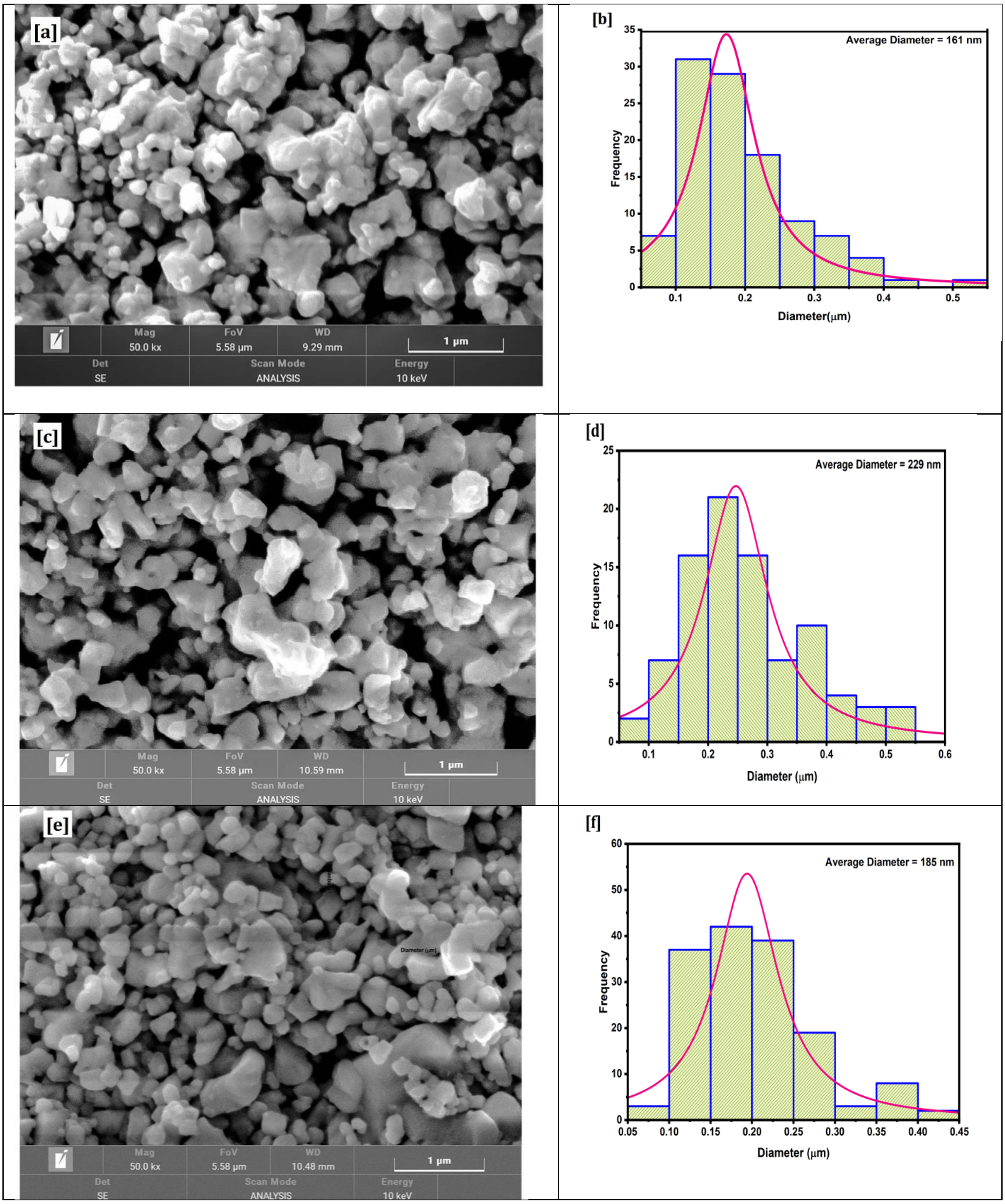
[a]
Mag 50.0 kx
FoV 5.58 μm
WD 9.29 mm
1 μm
Det SE
Scan Mode ANALYSIS
Energy 10 keV
[b]
Average Diameter = 161 nm
Frequency
Diameter(μm)
[c]
Mag 50.0 kx
FoV 5.58 μm
WD 10.59 mm
1 μm
Det SE
Scan Mode ANALYSIS
Energy 10 keV
[d]
Average Diameter = 229 nm
Frequency
Diameter (μm)
[e]
Mag 50.0 kx
FoV 5.58 μm
WD 10.48 mm
1 μm
Det SE
Scan Mode ANALYSIS
Energy 10 keV
[f]
Average Diameter = 185 nm
Frequency
Diameter (μm)

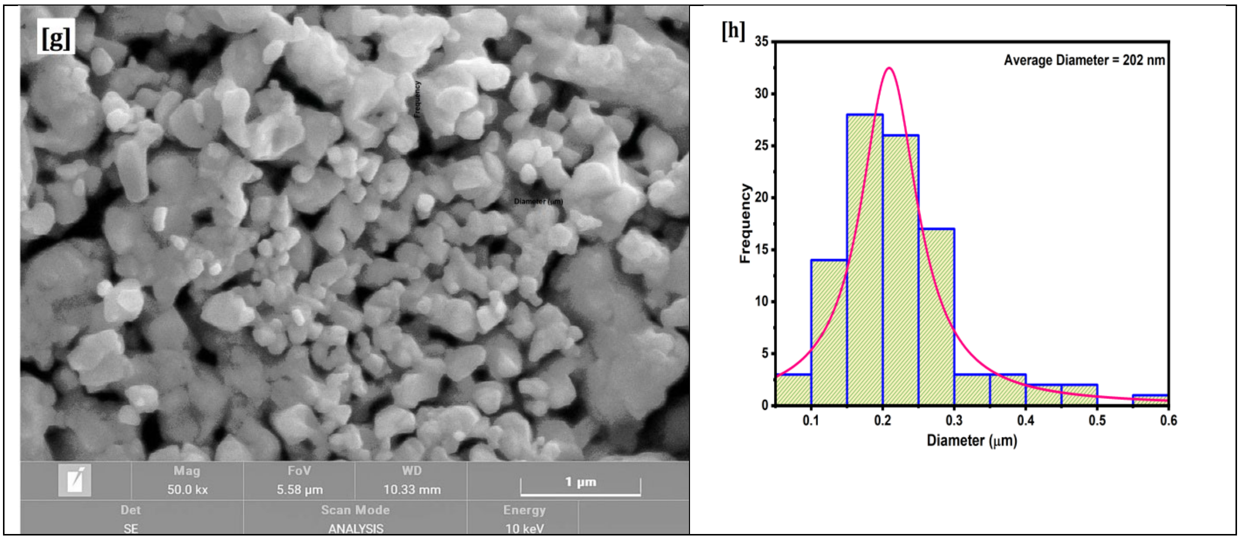

Figure S2. FE-SEM micrographs of and particle size distribution (a, b) $BaTiO_3$ (c, d) $BaTiO_3$:0.5%Eu (e, f) $BaTiO_3$:1.5%Eu (g, h) $BaTiO_3$:2.5%Eu.
